# Prediction accuracy of standard and total keratometry by swept-source optical biometer for multifocal intraocular lens power calculation

**DOI:** 10.1038/s41598-021-84238-1

**Published:** 2021-02-26

**Authors:** Hun Lee, Jae Lim Chung, Young Jun Kim, Jae Yong Kim, Hungwon Tchah

**Affiliations:** 1 Department of Ophthalmology, Asan Medical Center, University of Ulsan College of Medicine, 88, Olympic-Ro 43-Gil, Songpa-Gu, Seoul, 05505 South Korea; 2 Eyejun Ophthalmic Clinic, Seoul, Korea

**Keywords:** Diseases, Medical research

## Abstract

We aimed to compare the refractive outcomes of cataract surgery with diffractive multifocal intraocular lenses (IOLs) using standard keratometry (K) and total keratometry (TK). In this retrospective observational case series study, a total of 302 patients who underwent cataract surgery with multifocal IOL implantation were included. Predicted refractive outcomes were calculated based on the current standard formulas and a new formula developed for TK using K and TK, which were obtained from a swept-source optical biometer. At 2-month postoperatively, median absolute prediction errors (MedAEs) and proportion of eyes within ± 0.50 diopters (D) of predicted postoperative spherical equivalent (SE) refraction were analyzed. There was no significant difference between MedAEs or proportion of eyes within ± 0.50D of predicted refraction from K and TK in each formula. In TFNT00 and 839MP IOL cases, there was no difference between MedAEs from K and TK using any formula. In 829MP IOL cases, MedAE from TK was significantly larger than that from K in Barrett Universal II/Barrett TK Universal II (*P* = 0.033). In 677MY IOL cases, MedAE from TK was significantly larger than that from K in Haigis (*P* = 0.020) and Holladay 2 (*P* = 0.006) formulas. In the subgroup analysis for IOL, there was no difference between the proportion of eyes within ± 0.50 D of predicted refraction from K and TK using any formula. TFNT00 and 839MP IOLs were favorable with TK, with 677MY IOL with K and 829MP IOL being in a neutral position, which necessitates the study that investigates the accuracy of the new TK technology.

## Introduction

Techniques in cataract surgery have continuously evolved to improve the ability to fine-tune refractive outcomes with increasing prediction accuracy. Precise measurements of ocular parameters and ideal formula selection are essential to predict and achieve optimal refractive outcomes, specifically in cases of multifocal intraocular lens (IOL) implantation. The majority of the conventional keratometry and topography instruments assume a fixed relationship between the anterior and posterior corneal curvatures, thereby considering the cornea as a single refractive surface. Recently, the measurement of posterior corneal data and the application of these data to IOL calculation formulas have become a point of debate in cataract surgery with monofocal, multifocal, or toric IOL implantation^1–4^.

A novel optical biometer (IOLMaster 700; Carl Zeiss Meditec, Jena, Germany) that integrates swept-source optical coherence tomography (SS-OCT) and telecentric keratometry for ocular biometry has been recently introduced^5^. This new instrument can assess all the parameters that are required for IOL power calculation including standard keratometry (K), central corneal thickness (CCT), anterior chamber depth (ACD), lens thickness (LT), horizontal white-to-white (WTW) corneal diameter, and axial length (AL). The total keratometry (TK), integrated in the IOLMaster 700, is a new keratometry value combining telecentric three-zone keratometry and SS-OCT technology to determine anterior and posterior corneal surface measurements^5,6^.

Several articles have evaluated the refractive outcomes of cataract surgery with monofocal IOL implantation based on the K and TK data using the current standard formulas and new formulas developed for TK^1,2^. In comparison to the K data, a higher prediction accuracy of IOL power calculation was noted when using the TK data along with the new formula^1,2^. Even in patients who underwent previous laser refractive surgery, prediction accuracy can be improved by using the TK data with the new formula^3^. However, no study has evaluated the refractive outcomes of cataract surgery with multifocal IOL using the K and TK data. Therefore, this study aims to investigate the refractive outcomes of cataract surgery with currently introduced diffractive multifocal IOLs using the K and TK data in standard formulas (Haigis, SRK/T, Holladay 2, and Barrett Universal II) and new formulas developed for TK (Barrett TK Universal II).

## Results

**Figure 1:**
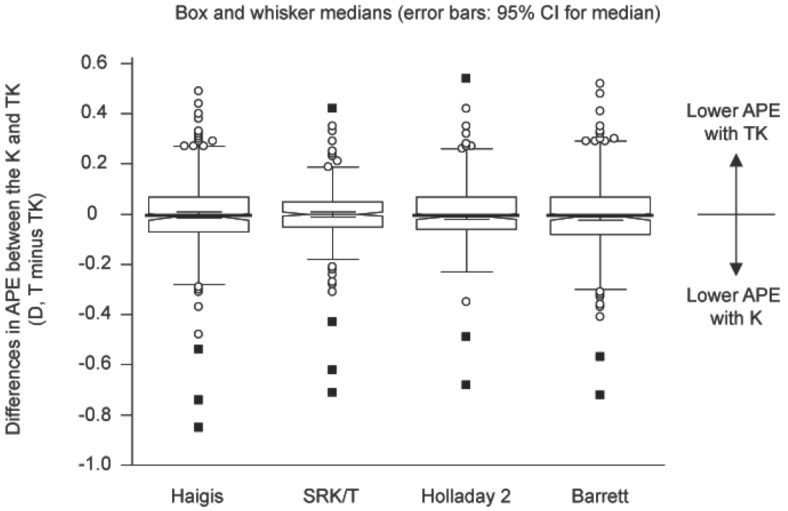
Box and whisker plot of the differences in the absolute prediction error between K and TK (K minus TK) using the Haigis, SRK/T, Holladay 2, and Barrett Universal II/ Barrett TK Universal II formulas. APE, absolute prediction error; TK, total keratometry; K, standard keratometry; D, diopters.

A total of 302 patients (302 eyes) who underwent cataract surgery with multifocal IOL implantation were enrolled in the study. Table 1 summarizes the patient demographics and ocular biometric characteristics. Final outcomes of the absolute prediction error (APE) calculated with the Haigis, SRK/T, Holladay 2, and Barrett Universal II/Barrett TK Universal II formulas based on the K and TK data are shown in Table 2. The mean APEs (MAEs) were defined as the mean absolute difference between the actual postoperative manifest refraction spherical equivalent (MRSE) and the predicted postoperative spherical equivalent (SE) refraction. The median APEs (MedAEs) were defined as the median absolute difference between the actual postoperative MRSE and predicted postoperative SE refractions. The MedAE from the TK data tended to be higher than that from the K data in the Haigis, Holladay 2, and Barrett Universal II/Barrett TK Universal II formulas, although the difference was not statistically significant (*P* = 0.664 for Haigis, *P* = 0.643 for Holladay 2, and *P* = 0.125 for Barrett Universal II/Barrett TK Universal II; Table 2 and Fig. 1). In case of the SRK/T formula, the MedAE from the TK data tended to be lower than that for the K data *(P* = 0.341). The Barrett Universal II formula showed the lowest MedAE among all formulas. However, there was no significant difference among the MedAEs from all formulas (*P* = 0.059).

**Table 1:** Patient demographics and ocular biometric characteristics.

**Parameter**	
Patients/eyes (n)	302/302
Right/left (%)	49.7/50.3
Male/female (%)	25.2/74.8
**Age (y)**	
Mean ± SD	57.74 ± 5.30
Median (range)	58.00 (46.00 to 72.00)
**Axial length (mm)**	
Mean ± SD	23.73 ± 1.17
Median (range)	23.46 (21.70 to 28.04)
**Anterior chamber depth (mm)**	
Mean ± SD	3.18 ± 0.34
Median (range)	3.15 (2.28 to 4.04)
**Standard keratometry**	
Mean ± SD	43.92 ± 1.30
Median (range)	44.00 (38.88 to 47.38)
**Manifest refraction sphere (D)**	
Mean ± SD	− 0.03 ± 2.31
Median (range)	0.63 (− 8.25 to 5.25)
**Manifest refraction cylinder (D)**	
Mean ± SD	− 0.70 ± 0.46
Median (range)	− 0.75 (− 2.00 to 0.00)
**IOL power**	
Mean ± SD	19.87 ± 3.27
Median (range)	20.50 (7.00 to 26.50)

SD, standard deviation; D, diopters; IOL, intraocular lens.

**Table 2:** Absolute prediction error between standard keratometry and total keratometry using the Haigis, SRK/T, Holladay 2, Barrett Universal II, and Barrett TK Universal II formulas.

	MAE	SD	MedAE	Minimum	Maximum	Percentage of eyes within diopter range indicated			
						± 0.25 D (%)	± 0.50 D (%)	± 0.75 D (%)	± 1.00 D (%)
Haigis T	0.267	0.217	0.220	0.00	1.43	56.3	87.1	97.7	98.7
Haigis TK	0.271	0.217	0.240	0.01	1.44	54.0	88.7	97.4	98.7
SRK/T T	0.258	0.206	0.218	0.00	1.35	57.9	88.4	97.0	99.3
SRK/T TK	0.266	0.208	0.213	0.00	1.36	56.6	89.1	96.7	99.3
Holladay 2 K	0.276	0.220	0.243	0.00	1.45	52.0	86.4	97.0	99.0
Holladay 2 TK	0.278	0.213	0.248	0.01	1.45	51.0	87.4	98.0	99.0
Barrett T	0.252	0.208	0.210	0.00	1.44	60.9	88.7	97.0	99.0
Barrett TK	0.262	0.212	0.215	0.00	1.44	57.9	86.4	97.0	99.3

MAE, mean absolute prediction error; MedAE, median absolute prediction error; D, diopters; K, keratometry; TK, total keratometry; SD, standard deviation.

**Figure 2:**
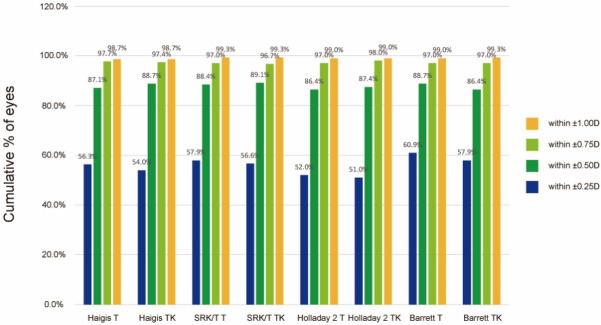
The cumulative percentage of eyes within the specified range of predicted postoperative spherical equivalent refraction outcomes for the different formulas including the Haigis, SRK/T, Holladay 2, and Barrett Universal II/Barrett TK Universal II formulas. D, diopters.

The proportions of eyes within ± 0.25 diopters (D), ± 0.50 D, ± 0.75 D, and ± 1.00 D of the predicted postoperative SE refraction outcome across all the formulas are shown in Fig. 2. The K group tended to show slightly higher proportion of eyes in the lower error ranges (within ± 0.25 D) than the TK group in all formulas applied. In the error range within ± 0.50 D, ± 0.75 D, and ± 1.00 D, both the K and TK groups showed comparable results (Fig. 2). There was no difference in the percentage of eyes within ± 0.50 D of predicted postoperative SE refraction outcomes between the K and TK data using each formula (*P* = 0.458 for Haigis, *P* = 0.804 for SRK/T, *P* = 0.664 for Holladay 2, and *P* = 0.265 for Barrett Universal II/ Barrett TK Universal II). eFigure 1 shows the stacked histogram comparing the percentage of eyes within a given diopter range of predicted SE refraction outcomes for overall cases.

**Figure 3:**
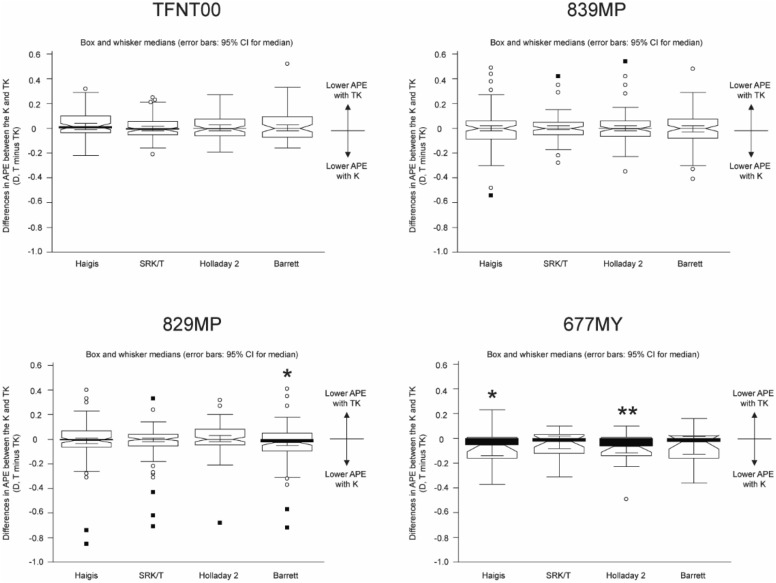
The box and whisker plots of the differences in the absolute prediction error between K and TK (K minus TK) using the Haigis, SRK/T, Holladay 2, and Barrett Universal II/Barrett TK Universal II formulas in each diffractive multifocal intraocular lens. APE, absolute prediction error; TK, total keratometry; K, standard keratometry; D, diopters. ^*^P < 0.05, ^**^P < 0.01.

Table 3 and Fig. 3 show the APE calculated with the Haigis, SRK/T, Holladay 2, and Barrett Universal II/Barrett TK Universal II formulas in each diffractive multifocal IOL:AcrySof IQ PanOptix (TFNT00; Alcon Laboratories, Inc., Fort Worth, TX, USA), AT LISA tri (839MP; Carl Zeiss Meditec), AT LARA (829MP; Carl Zeiss Meditec), and Bi-Flex M (677MY; Medicontur Medical Engineering Ltd., Zsámbék, Hungary). The target postoperative refraction was emmetropia in all eyes. In the TFNT00 IOL cases (n = 72), the MedAE from the TK data tended toward lower values compared to the K data, except in the case of the SRK/T formula, demonstrating the Barrett TK Universal II formula with TK as the lowest MedAE. In the 839MP cases (n = 96), the MedAE from the TK data tended toward lower values compared to the K data, except in the case of the Haigis formula, demonstrating the Barrett TK Universal II formula with the TK data as the lowest APE. In the 829MP IOL cases (n = 108), the MedAE from the TK data was larger than that from the K data in the Barrett Universal II/Barrett TK Universal II formula (*P* = 0.033). In the 677MY IOL cases (n = 26), the MedAE from the TK data was larger than that from the K data in the Haigis and Holladay 2 formulas (*P* = 0.020 for Haigis and *P* = 0.006 for Holladay 2; Fig. 3).

**Table 3:** Absolute prediction error between the standard keratometry and total keratometry using the Haigis, SRK/T, Holladay 2, Barrett Universal II, and Barrett TK Universal II formulas in each diffractive multifocal intraocular lens.

	MAE	SD	MedAE	Minimum	Maximum	Percentage of eyes within diopter range indicated			
						± 0.25 D (%)	± 0.50 D (%)	± 0.75 D (%)	± 1.00 D (%)
**TFNT00 (n = 72)**									
Haigis T	0.333	0.282	0.268	0.03	1.43	48.6	80.6	94.4	95.8
Haigis TK	0.297	0.270	0.260	0.01	1.44	48.6	87.5	95.8	95.8
SRK/T T	0.274	0.237	0.185	0.01	1.35	54.2	83.3	97.2	98.6
SRK/T TK	0.273	0.229	0.213	0.02	1.36	58.3	87.5	97.2	98.6
Holladay 2 K	0.280	0.27	0.228	0.00	1.45	54.2	84.7	93.1	97.2
Holladay 2 TK	0.271	0.254	0.210	0.01	1.45	58.3	88.9	95.8	97.2
Barrett T	0.265	0.283	0.170	0.00	1.44	65.3	83.3	93.1	95.8
Barrett TK	0.238	0.238	0.168	0.00	1.44	69.4	88.9	95.8	98.6
**839MP (n = 96)**									
Haigis T	0.240	0.177	0.200	0.00	0.69	60.4	88.5	100.0	100.0
Haigis TK	0.247	0.183	0.238	0.01	0.98	54.2	91.7	99.0	100.0
SRK/T T	0.243	0.166	0.200	0.00	0.81	58.3	92.7	97.9	100.0
SRK/T TK	0.234	0.154	0.200	0.00	0.76	62.5	94.8	99.0	100.0
Holladay 2 K	0.265	0.178	0.253	0.01	0.67	50.0	88.5	100.0	100.0
Holladay 2 TK	0.263	0.179	0.250	0.02	0.70	51.0	91.7	100.0	100.0
Barrett T	0.219	0.146	0.200	0.01	0.58	66.7	94.8	100.0	100.0
Barrett TK	0.220	0.151	0.198	0.01	0.66	61.5	93.8	100.0	100.0
**829MP (n = 108)**									
Haigis T	0.253	0.198	0.215	0.00	1.01	58.3	89.8	97.2	99.1
Haigis TK	0.267	0.212	0.215	0.01	1.06	59.3	88.9	96.3	99.1
SRK/T T	0.255	0.211	0.230	0.00	0.92	61.1	87.0	96.3	100.0
SRK/T TK	0.277	0.224	0.225	0.00	0.94	54.6	85.2	94.4	100.0
Holladay 2 K	0.294	0.227	0.250	0.00	1.10	50.9	83.3	96.3	99.1
Holladay 2 TK	0.289	0.215	0.255	0.01	1.09	49.1	84.3	97.2	99.1
Barrett T	0.265	0.199	0.233	0.00	0.84	56.5	85.2	97.2	100.0
Barrett TK	0.298	0.232	0.238	0.01	1.24	51.9	81.5	95.4	99.1
**677MY (n = 26)**									
Haigis T	0.250	0.188	0.235	0.00	0.71	53.8	88.5	100.0	100.0
Haigis TK	0.312	0.187	0.270	0.03	0.71	46.2	80.8	100.0	100.0
SRK/T T	0.284	0.233	0.235	0.00	1.16	53.8	92.3	96.2	96.2
SRK/T TK	0.325	0.248	0.313	0.00	1.11	38.5	88.5	96.2	96.2
Holladay 2 K	0.232	0.175	0.228	0.02	0.7	57.7	96.2	100.0	100.0
Holladay 2 TK	0.305	0.201	0.328	0.01	0.67	38.5	80.8	100.0	100.0
Barrett T	0.282	0.182	0.260	0.02	0.90	46.2	96.2	96.2	100.0
Barrett TK	0.339	0.204	0.333	0.00	0.84	38.5	73.1	96.2	100.0

MAE, mean absolute prediction error; MedAE, median absolute prediction error; D, diopters; K, keratometry; TK, total keratometry; SD, standard deviation.

There were differences among the APEs from all formulas in the TFNT00 and 839MP IOL cases (*P* = 0.011 for TFNT00 and *P* = 0.043 for 839MP). In case of the TFNT00 IOL, there was a difference in the APEs between the Haigis T and Barrett TK Universal II formulas (*P* = 0.011) and between the Haigis T and Barrett Universal II formulas (*P* = 0.025; eFigure 2). There was no difference among the APEs from all formulas in the 829MP and 677MY IOL cases (*P* = 0.113 for 829MP and *P* = 0.062 for 677MY).

**Figure 4:**
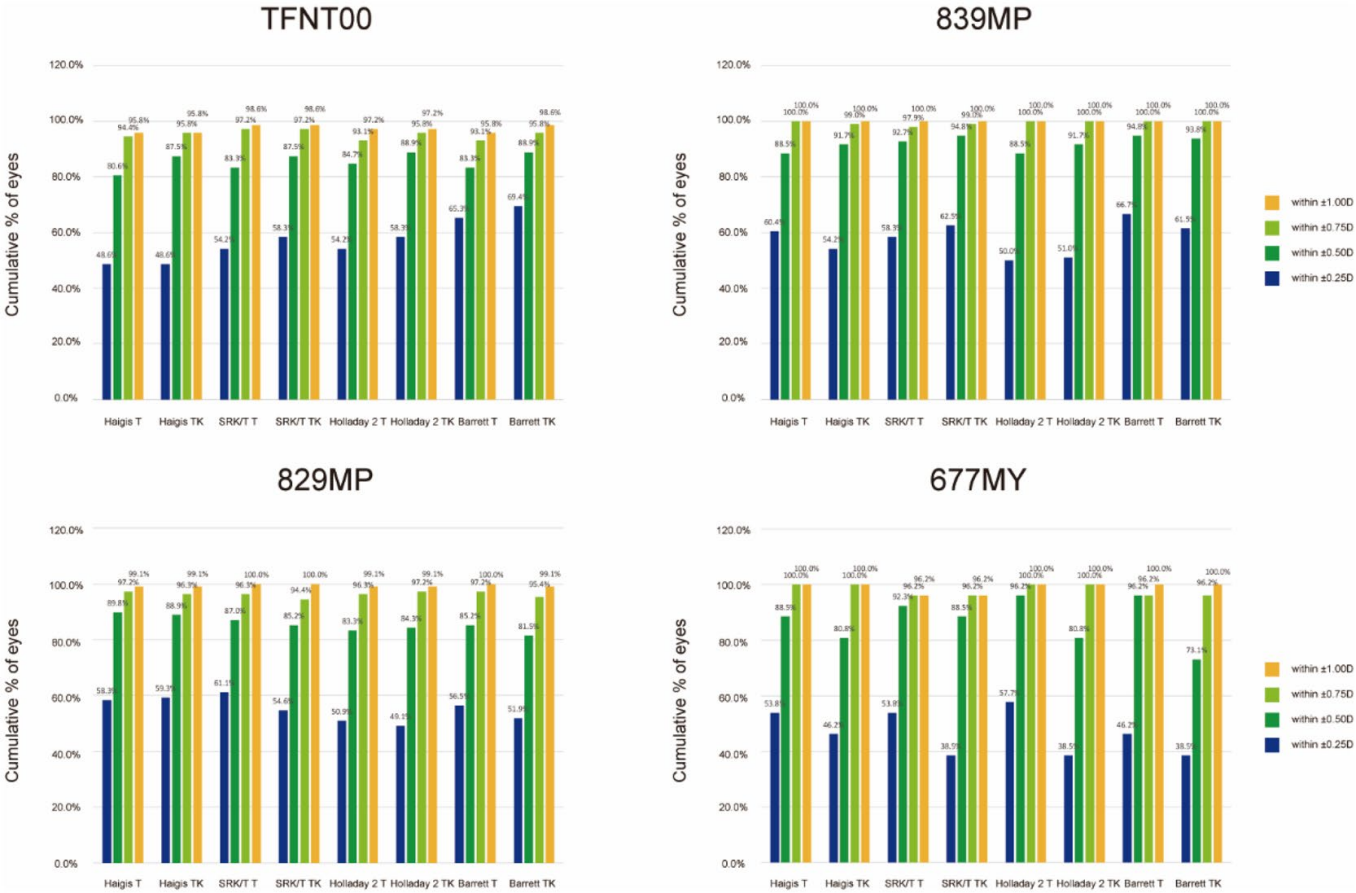
Cumulative percentage of eyes within the specified range of predicted postoperative spherical equivalent refraction outcomes for the different formulas including the Haigis, SRK/T, Holladay 2, and Barrett Universal II/Barrett TK Universal II formulas in each diffractive multifocal intraocular lens. D, diopters.

The proportions of eyes within ± 0.25 D, ± 0.50 D, ± 0.75 D, and ± 1.00 D of predicted postoperative SE refraction in each multifocal IOL are shown in Fig. 4. There was no difference in the percentage of eyes within ± 0.50 D of predicted postoperative SE refraction between the K and TK data using any formula in each IOL.

In case of the TFNT00 IOL, the TK group tended to show slightly higher proportion of eyes in all error ranges in all formulas applied (Fig. 4). In case of 677MY IOL, the TK group showed a slight trend toward lower percentage in the error range within ± 0.25 D, ± 0.50 D, and ± 0.75 D than the K group (Fig. 4). eFigure3 shows the stacked histogram comparing the percentage of eyes within a given diopter range of predicted SE refraction outcomes in each multifocal IOL.

## Discussion

In the present study, we investigated and compared the refractive outcomes of cataract surgery with multifocal IOL implantation using the K and TK data obtained from the IOLMaster 700 using current standard formulas (Haigis, SRK/T, Holladay 2, and Barrett Universal II) and new formulas developed for TK (Barrett TK Universal II). We found that the differences in APEs from the K and TK data vary according to the type of multifocal IOLs. Overall, in all diffractive multifocal IOL cases, we found that the MedAEs from the TK data were slightly higher than those from the K data in the Haigis, Holladay 2, and Barrett Universal II/Barrett TK Universal II formulas, whereas the MedAE from the TK data tended to be lower in the SRK/T formula. Specifically, in case of the TFNT00 and 839MP IOL, the MedAE from the TK data tended to be lower than that from the K data across all the formulas, except the SRK/T for TFNT00 IOL or the Haigis formula for 839MP IOL. Notably, in the 829MP IOL cases, the MedAE from the TK data was significantly higher than that from the K data in the Barrett Universal II/Barrett TK Universal II formula. Additionally, in the 677MY IOL cases, significantly higher MedAEs were noted from the TK data in the Haigis and Holladay 2 formulas.

After the introduction of the total corneal power, several previous studies have shown an improvement in the refractive outcomes of TK over K in conventional cataract surgery, whereas other studies did not show any benefits^7–9^. However, the total corneal power in the previous studies was measured using the Scheimpflug camera system for corneal topography. As the topography system applies its own algorithm for the calculation of the total corneal power, it could be possible to yield a different value between topography systems. In our study, using the new total corneal power derived by the SS-OCT-type optical biometer (IOLMaster 700) for the IOL calculation, we compared the refractive outcomes of cataract surgery with four diffractive multifocal IOLs using the K and TK data in the Haigis, SRK/T, Holladay 2, and Barrett Universal II/Barrett TK Universal II formulas. The TK values derived by the IOLMaster 700 are calculated using the data from both the anterior and posterior cornea and the corneal thickness.

Subgroup analyses showed that the TFNT00 and 839MP IOLs showed more favorable results with TK, the 677MY IOL showed favorable results with K, and the 829MP IOL was in a neutral position between K and TK. In one recent study comparing the refractive outcomes following conventional cataract surgery with IOL (601P/PY; Carl Zeiss Meditec) implantation using the K and TK data in the SRK/T, HofferQ, Haigis, Holladay 1, Holladay 2, Barrett, and Barrett TK Universal II formulas, TK had lower MAEs and MedAEs compared with K, demonstrating the Barrett TK Universal II formula with the lowest MAEs^1^. The proportions of eyes within ± 0.25, ± 0.50, and ± 1.00 D of predicted refraction were slightly higher using the TK data. The mean difference between K and TK was only 0.03 D (K 44.56 ± 1.18 D and TK 44.59 ± 1.22 D), showing very good agreement, with an intraclass correlation coefficient of 0.99^1^. Another study regarding cataract surgery with IOL (CT Asphina 409 M/MP; Carl Zeiss Meditec) implantation demonstrated that the APE calculated from the TK data tended toward lower values compared to that from the K data in the Haigis and Barrett Universal II/Barrett TK Universal II formulas^2^. The author concluded that a higher prediction accuracy can be expected using the TK data along with the above two formulas.

Unlike those two studies, this study is based on the refractive outcomes from cataract surgery with diffractive multifocal IOL implantation. According to literature, the application of the TK data can be particularly advantageous for certain cases of higher astigmatism and post-refractive surgery cases^3,10,11^. In cataract surgery with toric IOL implantation, the selection of toric IOLs based on the anterior and posterior corneal measurements is mandatory to prevent postoperative overcorrection or undercorrection^11–13^. Moreover, small values of APE for SE and cylinder from the TK data were more frequent than from the K data, and calculation of the differences of APE between the K and TK data confirmed more accurate results of the TK, specifically for cylinder outcomes of the Haigis-T formula^2^. Moreover, it is notable that TK considering corneal thickness and posterior corneal curvature can be advantageous with additional accurate information about total corneal power in eyes that have undergone post-refractive surgery^3,10,14^. Upon comparison of the prediction accuracy of IOL power calculation methods after previous laser refractive surgeries using the K and TK data, the Barrett True-K formula using the TK data provided the lowest mean refractive prediction error and median absolute error for eyes that have undergone prior myopic and hyperopic laser refractive surgery^3^. Additionally, another study reported the TK data as a valid option within the standard Haigis formula in a group of post-refractive cataract patients^10^.

According to the results from overall cases, the Barrett Universal II formula showed the lowest MedAE among all formulas. In cases of the TFNT00 and 839MP IOLs, the Barrett TK Universal II formula showed the lowest MedAE among all formulas. In cases of the 829MP and 677MY IOLs, the performance of the Barrett TK Universal II formula was not as good as in the other IOLs. The newly introduced Barrett TK Universal II formula was designed to be used with TK. Therefore, it could be reasonable that the lowest APEs are shown with the TK data using the Barrett TK Universal II formula. However, it is important that the currently available IOL calculation formulas have been based on the K data, instead of the TK data obtained from the IOLMaster 700, thereby necessitating the accumulation of huge data for refractive outcomes based on the TK data. Additionally, there could be a few conflicting results regarding the APE because of the characteristics of each diffractive multifocal IOL, specifically 677MY and 829MP IOLs showing the favorable results with K in a certain formula. Future studies involving a larger number of patients and a variety of IOL models are required to further confirm the accuracy of the Barrett TK Universal II formula using the TK data.

The limitation of this study is that it is retrospective in nature. As all the cases were confined within the normal range of all parameters, a surgeon should be cautious when applying these results to eyes in extreme parameters related to very long or short AL. However, our study provides notable data on a large patient case series of cataract surgery with four kinds of diffractive multifocal IOL implantation. Greater numbers with TK data, posterior corneal values, and representative formulas remain desirable to support the current findings. Further prospective studies with a large number of patients evaluating the refractive outcomes of cataract surgery with varied multifocal IOLs using the K and TK data in all current formulas, even in eyes with higher astigmatism and post-refractive surgery status, are required to investigate the accuracy of the new TK technology.

In conclusion, this study demonstrated that the differences between APEs from the K and TK data vary according to the type of multifocal IOLs. Subgroup analysis upon specific IOL types showed that the TFNT00 and 839MP IOLs were favorable with the TK, the 677MY IOL was favorable with the K, and the 829MP IOL was in a neutral position between K and TK. Cataract surgeons should keep in mind that the comparisons between the application of the K and TK data to multiple IOL calculation formulas are crucial to prevent unwanted postoperative refractive error in cataract surgery with monofocal, multifocal, or toric IOL implantation.

## Methods

This study was conducted with the approval of the Institutional Review Board of the Asan Medical Center and the University of Ulsan College of Medicine, Seoul, South Korea (2020–1290). The study adhered to the tenets of the Declaration of Helsinki and followed good clinical practice guidelines. Informed consent was obtained from all patients after explanation of the purpose and possible consequences of the study.

This retrospective observational case series study included 302 patients (302 eyes) who underwent cataract surgery with diffractive multifocal IOL implantation. The inclusion criteria included the completion of preoperative biometry and keratometry assessment with the IOLMaster 700 and successful cataract surgery with corrected distance visual acuity (CDVA) of at least logMAR 0.1 at 2 months following surgery. Patients were excluded if they had undergone previous ocular surgery, experienced trauma, or had other existing ocular pathologies other than cataract. For patients undergoing bilateral cataract surgery, one eye from each patient was randomly selected for inclusion in the analysis.

Each patient underwent routine preoperative assessments for cataract removal and IOL placement with the IOLMaster 700. All optical biometric parameters including conventional K, TK, posterior keratometry, CCT, WTW corneal diameter, ACD, LT, and AL were measured. To ensure the repeatability of measurements, parameters were evaluated twice by an experienced technician. TK is compatible with keratometry data, allowing the TK values to be included in classic IOL calculation formulas^2^. The IOL power calculations were provided by the IOLMaster 700 unit (Barrett Suite, Software Version 1.80.6.60340). For consistency, the IOL power was subsequently calculated using optimized constants from the User Group for Laser Interference Biometry website.

K (K group) and TK (TK group) were used for IOL power calculation in current standard formulas (Haigis, SRK/T, Holladay 2, and Barrett Universal II) and new formulas developed for TK (Barrett TK Universal II). Emmetropic IOL power and predicted refractive outcomes were calculated using K and TK in all formulas using the IOLMaster 700. Following the completion of preoperative assessment and measurements, all patients underwent scheduled cataract surgery, performed by a single, experienced surgeon. After the administration of topical anesthesia (0.5% proparacaine hydrochloride), the phacoemulsification surgery was performed. A continuous curvilinear capsulorrhexis marker with a diameter of 6.0 mm was used to reference the corneal plane. The main clear corneal incision was made using a 2.2-mm keratome, followed by capsulorrhexis using a capsulorrhexis forceps. Following hydrodissection, phacoemulsification of the nucleus and cortical aspiration were performed using a phacoemulsifier (Centurion; Alcon Laboratories, Inc.). Subsequently, the multifocal IOL was implanted into the capsular bag of the eye. The surgeons selected the type of IOL to be implanted based on personal preference and patient suitability. The multifocal IOLs used in the present study were as follows: TFNT00 (Alcon Laboratories, Inc.), 839MP (Carl Zeiss Meditec), 829MP (Carl Zeiss Meditec), and 677MY (Medicontur Medical Engineering Ltd.). The target postoperative refraction was emmetropia in all eyes. No intraoperative complications were observed.

The postoperative examinations were performed at 2 months following surgery and included autorefraction (RK-F2 Full Auto Ref-Keratometer, Canon, Tokyo, Japan), manifest refraction, and CDVA. The evaluation was performed using the APEs and the proportion of eyes within ± 0.25 D, ± 0.50 D, ± 0.75 D, and ± 1.00 D of predicted postoperative SE refraction outcomes. The mean APEs (MAEs) were defined as the mean absolute difference of the actual postoperative MRSE refraction and the predicted postoperative SE refraction. The median APEs (MedAEs) were defined as the median absolute value of the difference between the actual postoperative MRSE refraction and the refractive error predicted by the Haigis, SRK/T, Holladay 2, and Barrett Universal II/Barrett TK Universal II formulas^15^.

### Statistical analyses

Wilcoxon signed-rank tests were used to evaluate the differences between the APEs obtained from the K and TK data using each formula. The APEs from the K and TK data of all formulas were compared using the Friedman test with Bonferroni post-hoc correction for multiple comparisons. The McNemar's test was performed to compare the percentage of eyes within ± 0.50 D of the APE between the K and TK data in each formula. All the data were statistically analyzed using the SPSS software version 25.0 (IBM, Armonk, NY, USA). A *P* value of less than 0.05 was considered statistically significant.

## Supplementary Information

Supplementary Legends.

Supplementary Information 1.

Supplementary Information 2.

Supplementary Information 3.

## Data Availability

The data sets generated and analysed during the current study are available from the corresponding author on reasonable request.
