# Supplementary material for: Prediction accuracy of standard and total keratometry by swept-source optical biometer for multifocal intraocular lens power calculation
Source: Sci Rep. 2021 Feb 26;11:4794. doi: 10.1038/s41598-021-84238-1 (PMC7910298; doi:10.1038/s41598-021-84238-1)
Supplement: Supplementary file 1 — Supplementary Legends. [file 41598_2021_84238_MOESM1_ESM.docx]

**Online-Only Figures**

**eFigure 1**. Stacked histogram comparing the percentage of eyes within the specified range of predicted postoperative spherical equivalent refraction outcomes.

**eFigure 2**. The box and whisker plots of the absolute prediction error between K and TK using the Haigis, SRK/T, Holladay 2, and Barrett Universal II/Barrett TK Universal II formulas in TFNT00 multifocal intraocular lens. TK = total keratometry, K = standard keratometry, D = diopters.

**eFigure 3**. Stacked histogram comparing the percentage of eyes within the specified range of predicted postoperative spherical equivalent refraction outcomes in each diffractive multifocal intraocular lens.
