# Supplementary figures and images for: Prediction accuracy of standard and total keratometry by swept-source optical biometer for multifocal intraocular lens power calculation
Source: Sci Rep. 2021 Feb 26;11:4794. doi: 10.1038/s41598-021-84238-1 (PMC7910298; doi:10.1038/s41598-021-84238-1)

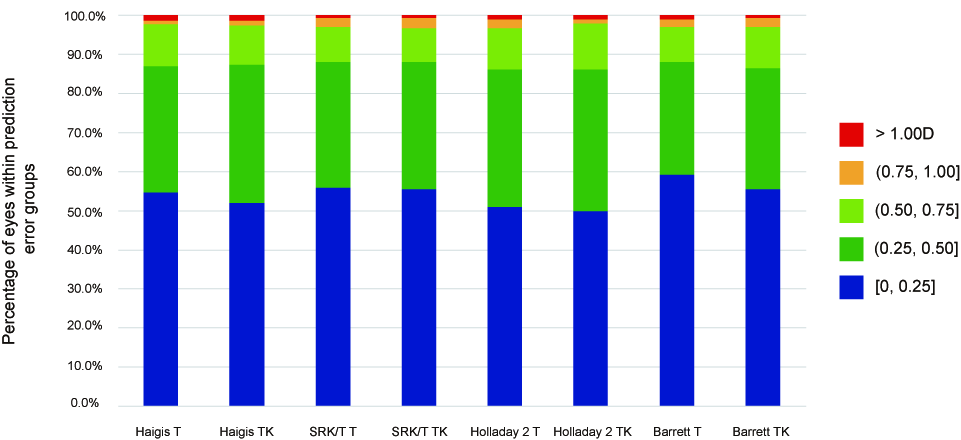

Supplement: Supplementary file 2 — Supplementary Information 1. [file 41598_2021_84238_MOESM2_ESM.tif]

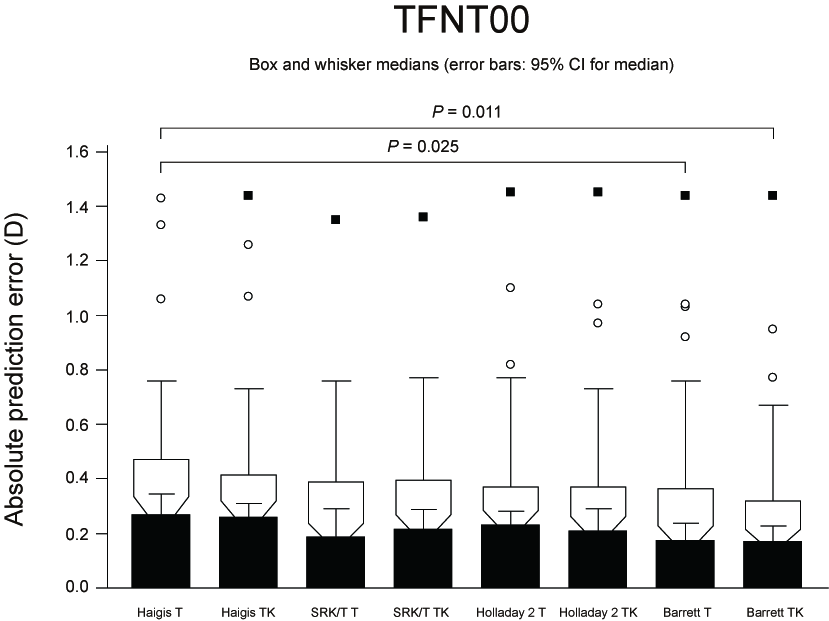

Supplement: Supplementary file 3 — Supplementary Information 2. [file 41598_2021_84238_MOESM3_ESM.tif]

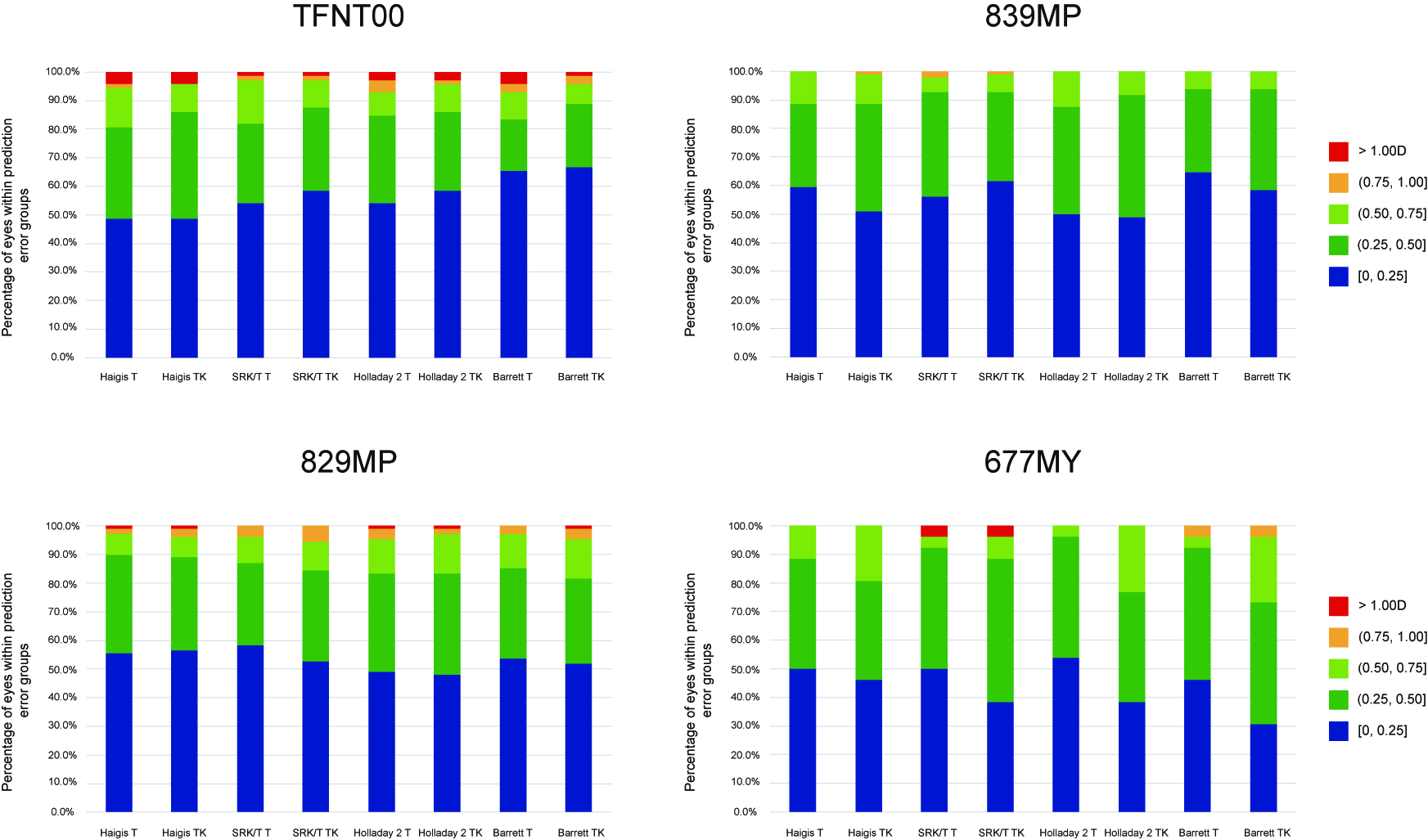

Supplement: Supplementary file 4 — Supplementary Information 3. [file 41598_2021_84238_MOESM4_ESM.tif]
